# Association of psychotropic medications with the use of coercive measures and recidivism during forensic psychiatric care: a Swedish nationwide register-based study

**DOI:** 10.1136/bmjment-2026-302515

**Published:** 2026-06-17

**Authors:** Taalke Maria Sitter, Suvi Savinen, Peter Andiné, Anja Fernqvist, Hanna Edberg, Isabell Brikell, Brian M D’Onofrio, Tatja Hirvikoski, Ralf Kuja-Halkola, Agnieszka Szwajda, Seena Fazel, Thomas Nilsson, Zheng Chang

**Affiliations:** 1 Department of Medical Epidemiology and Biostatistics, Karolinska Institutet, Stockholm, Sweden; 2 School of Educational Sciences and Psychology, University of Eastern Finland, Joensuu, Finland; 3 Center for Ethics, Law and Mental Health, Department of Psychiatry and Neurochemistry, Institute of Neuroscience and Physiology, Sahlgrenska Academy, University of Gothenburg, Gothenburg, Sweden; 4 Department of Forensic Psychiatry, Sahlgrenska University Hospital, Gothenburg, Sweden; 5 Department of Forensic Psychiatry, National Board of Forensic Medicine, Stockholm, Sweden; 6 Swedish Prison and Probation Service, Norrköping, Sweden; 7 Department of Biomedicine, Aarhus University, Aarhus, Denmark; 8 Department of Psychological and Brain Sciences, Indiana University Bloomington, Bloomington, Indiana, USA; 9 Centre for Psychiatry Research, Region Stockholm, Stockholm, Sweden; 10 Department of Women’s and Children’s Health, Karolinska Institutet, Stockholm, Sweden; 11 Department of Psychiatry, University of Oxford, Oxford, UK

**Keywords:** Psychiatry, Psychopharmacology

## Abstract

**Background:**

Patients in forensic psychiatric care (FPC) are often treated with various psychotropic medications. However, evidence regarding their effectiveness in this setting is lacking.

**Objective:**

The study aimed to estimate how different psychotropic medications are associated with the use of coercive measures and recidivism during FPC.

**Methods:**

In this observational national register-based study, we included all patients newly registered as admitted to FPC in Sweden between January 2009 and August 2024. Exposures included major classes of psychotropic medication (typical antipsychotics, atypical antipsychotics, antidepressants, hypnotics and sedatives, antiepileptic medications, opioids, medications for addictive disorders and mood stabilisers), antipsychotic treatment strategies (no use, monotherapy, polypharmacy, long-acting injectables or clozapine) and specific antipsychotic agents. Outcomes were the use of coercive measures and recidivism into criminal behaviour. We performed a within-individual analysis using conditional generalised estimating equation models, where the risk of outcomes was compared between exposed and non-exposed time periods.

**Findings:**

In total, 2690 patients were included, of which 25.1% experienced at least one occasion of coercion and 27.1% at least one event of recidivism during FPC. Atypical antipsychotics were associated with a reduced risk of subsequent use of coercive measures (OR 0.70, 95% CI 0.55 to 0.89) and recidivism (OR 0.79, 95% CI 0.63 to 0.99). Medication for addictive disorders was associated with a reduced risk of coercive measures by 34% (OR 0.66, 95% CI 0.47 to 0.93). Among antipsychotic agents, clozapine was associated with the largest risk reduction in the use of coercive measures (OR 0.48, 95% CI 0.33 to 0.69) and recidivism (OR 0.49, 95% CI 0.33 to 0.74). There was no significant difference between long-acting injectable, oral monotherapy or polypharmacy.

**Conclusions:**

We observed that atypical antipsychotics, especially clozapine, were associated with a risk reduction for coercive measures and recidivism during FPC. Additionally, medications for addictive disorders were associated with a reduced risk of coercive measures.

**Clinical implications:**

Our results can help inform decision-making processes in this clinical setting regarding pharmacotherapy but should be interpreted as one of many aspects influencing treatment success in FPC.

**WHAT IS ALREADY KNOWN ON THIS TOPIC**

- There is a lack of evidence on the effectiveness of pharmacotherapy in forensic psychiatric care, specifically regarding outcomes relevant to the setting such as reduction of coercive measures and recidivism.

**WHAT THIS STUDY ADDS**

- To our knowledge, this is the largest study on the association of pharmacotherapy and outcomes relevant for forensic psychiatric care, as well as the first to specifically investigate the use of coercive measures to date.
- Using national registry data and a self-controlled design, we found that atypical antipsychotic use was associated with a reduced risk of subsequent coercive measures and recidivism in comparison to non-usage. Further, medication for addictive disorders was associated with a reduced risk for the use of coercive measures.
- Clozapine was associated with the biggest risk reduction of coercive measures and recidivism, followed by olanzapine.
- No difference was detectable between long-acting injectable and oral antipsychotics in monotherapy and polypharmacy.

**HOW THIS STUDY MIGHT AFFECT RESEARCH, PRACTICE OR POLICY**

- The results of this study can support clinicians making treatment decisions regarding pharmacotherapy for patients in forensic psychiatry, as well as inform treatment guidelines for this setting.

## Introduction

 The population of forensic psychiatric patients is characterised by severe mental disorders combined with a history of criminal behaviour. Accordingly, the goals of treatment in forensic psychiatric care (FPC) include both management of psychiatric disorders as well as reducing the risk of subsequent criminal behaviour. A wide range of psychotropic medications, especially antipsychotics, are commonly used to achieve these aims. Previous studies have reported high levels of antipsychotic use, including off-label antipsychotics and antipsychotic polypharmacy, as well as general psychotropic polypharmacy.^1 2^ However, a systematic review highlighted a lack of high-quality evidence regarding pharmacotherapy in the context of FPC,^3^ and there have been repeated calls for more rigorous evidence in forensic psychiatry.^4 5^ While many psychotropic treatment regimens have been evaluated in general psychiatry settings for outcomes like symptom reduction, adverse effects or antisocial behaviour,^6^,^8^ evidence on outcomes specific to FPC is scarce. Key outcomes of relevance include the use of coercive measures and recidivism during treatment. Both can serve as proxies for treatment success and may influence whether discharge is appropriate, or if FPC should be extended.

Coercive measures can be defined as measures used against the patient’s will. This can include a restriction of electronic communication, involuntary treatment and limiting a person’s freedom of movement (eg, physical restraint or seclusion).^9^ These measures, which severely restrict patient autonomy, are used with the rationale that they prevent the patient from (further) harming themselves or others in acute risk situations. However, use of such measures presents ethical and legal challenges, as it compromises the autonomy of the patient and can lead to adverse outcomes, such as post-traumatic stress disorder and long-term deterioration of mental health.^9 10^ Reducing the use of coercive measures could benefit both patients and staff and is supported by the European Psychiatric Association.^5^ Recidivism during care is of interest, as the reduction of subsequent criminal behaviour is a primary goal of FPC. Criminal behaviours during FPC include possession and distribution of illegal substances, violent acts and threats, theft, absconding and more.^11^ Relapse into criminal behaviour during care indicates that treatment goals have not been fully achieved, and treatment strategies might need to be revised.

## Objective

The present study aims to estimate to what extent different psychotropic medications are associated with the use of coercive measures and criminal recidivism during FPC. While randomised controlled trials are considered the gold standard in evaluating the effectiveness of pharmacological treatment, their application in forensic psychiatry is limited by both ethical and practical challenges. Therefore, we analysed observational data from Swedish national registries using a self-controlled design to minimise confounding.^12^

## Methods

### Study design/setting

This register-based cohort study used data from the Swedish National Forensic Psychiatric Register (SNFPR/RättspsyK), which is a nationwide register that collects information on patients sentenced to FPC in Sweden since 2009, with a yearly coverage ranging from 84% to 96%.^13 14^ It contains demographic, criminological, psychiatric and treatment-relevant information for every patient at treatment start (new registration), yearly follow-up, transfer between clinics and end of care (discharge or death). Data are typically collected in a yearly interval, which in this study will be referred to as ‘data points’. The time between two data entries will be referred to as ‘periods’. In Sweden, a person who has committed a criminal offence may be sentenced to FPC instead of prison if the court, following a forensic psychiatric assessment, concludes that the individual suffers from a severe mental disorder.^15^ In the Swedish legislation, ‘severe mental disorder’ is a medicolegal term that typically involves consideration of both the type and severity of the disorder. Every year, approximately 300 individuals are sentenced to FPC in Sweden,^16^ and the median length of care is 7.5 years, including inpatient and outpatient periods.^17^

### Participants

All individuals in the SNFPR sentenced and admitted to FPC after 1 January 2009 with at least two data entries by 31 August 2024 were included in the study. Individuals were excluded if the date of treatment start was missing or if they entered SNFPR with a different data entry than ‘new registration’. Individuals with multiple sentences to FPC during the study period were included with every sentence that had at least two corresponding data points in the SNFPR.

### Measurements

The exposures of interest were pharmacological treatments during FPC. Medication use was measured at every data point and included all standing prescriptions at the given time, as well as pro re nata medications that had been given on more than three occasions in the prior week. First, we assessed the use of major classes of psychotropic medication, which include typical and atypical antipsychotics (Anatomical Therapeutic Chemical code: N05A, except N05AN01; see online supplemental eTable 1), antidepressants (N06A), attention-deficit hyperactivity disorder (ADHD) medication (N06B), hypnotics and sedatives (N05B and N05C), antiepileptic medications (N03A, except N03AG01 and N03AF01), opioids (N02A, except N02AE01), medications for addictive disorders (N07B and N02AE01) and mood stabilisers (N05AN01, N03AG01, N03AF01). This exposure was treated as binary (yes/no) for every major psychotropic medication at every data point.

Second, antipsychotic treatment strategies were classified for each data point into five mutually exclusive categories, similar to a previous study^18^: (1) no usage, (2) oral monotherapy (non-clozapine oral antipsychotic monotherapy), (3) oral polypharmacy (non-clozapine oral antipsychotic polypharmacy), (4) long-acting injectables (LAI) with or without concomitant non-clozapine oral antipsychotic use and (5) clozapine (clozapine with or without concomitant other antipsychotics).

Third, we further examined specific antipsychotic agents, including the five most frequently used ones—olanzapine (N05AH03), aripiprazole (N05AX12), clozapine (N05AH02), quetiapine (N05AH04) and risperidone (N05AX08)—as well as other atypical antipsychotics and typical antipsychotics (online supplemental eTable 1). This exposure was treated as binary (yes/no) for every antipsychotic at every data point.

Outcomes of interest were the use of coercive measures or any form of criminal recidivism. Use of coercive measures was defined as use of physical restraint, seclusion or medication administration under physical restraint. This outcome can only occur during inpatient care periods of FPC. Periods that an individual has exclusively spent in outpatient care were therefore not considered eligible for the analysis of this outcome. Recidivism was defined as any behaviour during FPC that has led or could lead to a police report. Conviction information was not considered. Recidivism could take place during inpatient and outpatient periods. Both outcomes were recorded in SNFPR at every data point, except at admission/new registration, and captured any occurrence of the respective outcome since the last data point (yes/no) (online supplemental eFigure 1).

SNFPR records up to four diagnoses at every data point. For the description of the sample, we reported the proportion of schizophrenia spectrum disorder (International Classification of Diseases-10th Revision code: F20–F29), substance use disorder (SUD) (F1*.2, F1*.7), bipolar disorder (F31), recurrent depressive disorder (F33), ADHD (F90), disorders of adult personality and behaviour (F60–F69), autism spectrum disorder (F84.0, F84.1, F84.5, F84.8 and F84.9) and intellectual disability (F70–F79), recorded at any time during FPC.

### Statistical methods

We used a self-controlled design to examine the association between the exposure of interest and the outcomes.^12^ A period was defined as exposed if it followed a data point with the respective medication. The subsequent data point was used to determine the outcome for that period, as the outcome measures gave information on the time since the last data point. This staggered design ensured temporal precedence of the exposure relative to the outcome, reducing the risk of reversed causation (online supplemental eFigure 1).

We used a conditional generalised estimating equation model with a logit link to estimate ORs with 95% CIs. Each individual was entered as a separate cluster, meaning that every individual served as their own control by comparing periods of exposure and non-exposure. This design accounts for all confounders that are constant within a person throughout the study period (eg, genetic characteristics, chronic comorbidities). All models are further adjusted for time in care, measured in full years since treatment start. Individuals with multiple sentences to FPC during the study period were analysed across their FPC sentences, so that all observed periods of an individual within the study period were compared with each other.

For non-mutually exclusive exposures (classes of major psychotropic medications and specific antipsychotic agents), estimates reflect the odds of outcomes between exposed and unexposed periods of the specific medication, while adjusted for other medications and time in care. In contrast, for models using mutually exclusive exposure categories (antipsychotic treatment strategies), the estimates represent the odds of the outcome during periods with different treatment strategies, compared with periods with oral monotherapy, adjusted for time in care.

We performed subgroup analyses for patients with and without SUD, as it presents a common comorbidity in FPC, which potentially complicates treatment approaches. The outcome criminal recidivism was also analysed separately for inpatient and outpatient periods. A sensitivity analysis excluding the first period after admission for every patient was conducted, as the year after admission presents a time with more medication changes than later treatment periods,^1^ which increases the risk of exposure misclassification. Further, prescriptions might more often be of a reactive nature in regard to the analysed outcomes during the early treatment time, which potentially violates the model assumptions of outcomes not influencing subsequent exposures.^12^

Only periods with non-missing information about medication and outcome were considered in the analysis (online supplemental eMethods 1). All analyses were performed with the package drgee in R.^19^ The reporting of this study followed the Reporting of Studies Conducted Using Observational Routinely Collected Health Data for Pharmacoepidemiology.

## Findings

In total, 2690 individuals with 2742 sentences to FPC fulfilled the inclusion criteria and contributed with 13 318 eligible observation periods to the analysis. The study sample was predominantly male (82.7%), with a median age of 34 years at admission, and the median length of observed treatment per sentence was 4.26 years. The most common psychiatric disorder was schizophrenia spectrum disorder (77.4%), followed by SUD (36.0%) and personality disorder (26.0%). Further characteristics can be found in table 1. Among the included patients, 674 (25.1%) experienced a coercive measure at least once during the study period, of which seclusion was the most common type, with 614 patients experiencing it at least once, followed by medication administration under physical restraint (278 patients) and general physical restraint (272 patients, online supplemental eTable 2). Further, 730 (27.1%) had at least one reported recidivism event. Among those patients, 317 (43.4%) had at least one incident of violent recidivism, and 278 (38.1%) drug-related recidivism (online supplemental eTable 2). The total number of coercive measures and recidivism by observed treatment periods is presented in online supplemental eTable 3.

**Table 1:** Sample characteristics of patients in forensic psychiatry care

	Sample
Total number of individuals	2690
Total number of sentences	2742
Sex, male, n (%)	2225 (82.71)
Age at admission in years, median (IQR)*	34 (27–45)
Length of observed treatment in years, median (IQR)*	4.26 (2.48–7.11)
Place of birth, Sweden, n (%)	1736 (64.54)
Diagnoses	
Schizophrenia spectrum disorder	2083 (77.43%)
Substance use disorder	968 (35.99%)
Bipolar disorder	229 (8.51%)
Recurrent depressive disorder	71 (2.64%)
ADHD	414 (15.39%)
Disorders of adult personality and behaviour	700 (26.02%)
Autism	522 (19.41%)
Intellectual disability	436 (16.21%)
Outcome during study period (≥1 occurrence)	
Use of coercive measures	674 (25.06%)
Any recidivism	730 (27.14%)

* Analysis unit: sentences.

ADHD, attention-deficit hyperactivity disorder.

### Major classes of psychotropic medication

**Figure 1:**
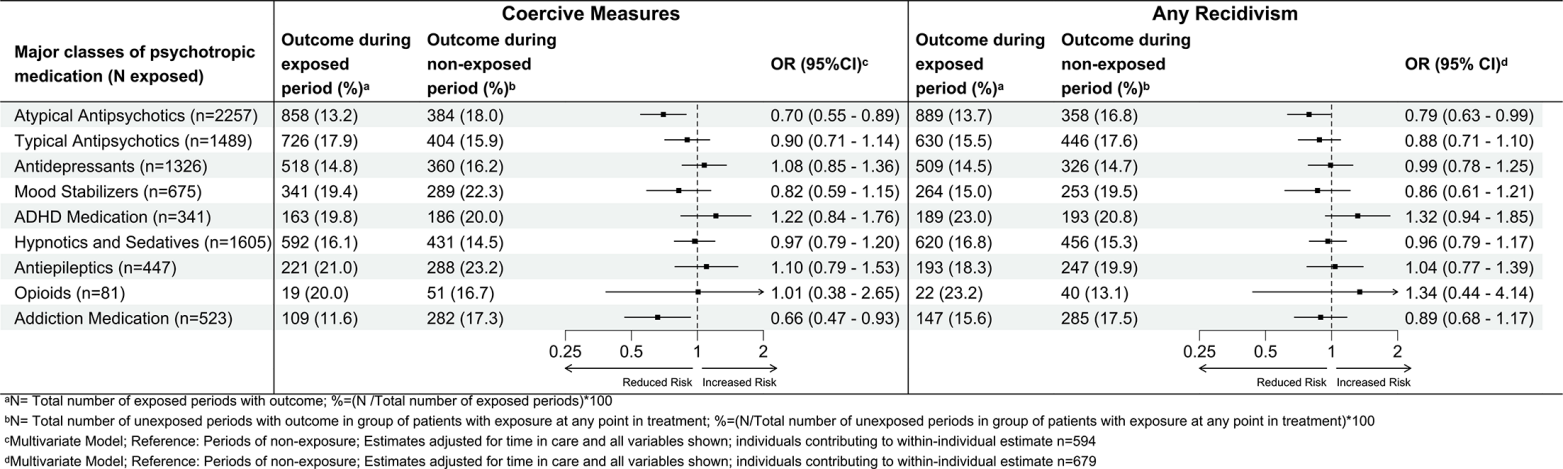
Associations between major psychotropic medication and coercive measures and recidivism. ADHD, attention-deficit hyperactivity disorder.

Atypical antipsychotics were the most commonly used psychotropic medication class, with 2257 individuals (84.0%) receiving them at least once during care. The second and third most commonly used psychotropic medications were hypnotics and sedatives (59.7%) and typical antipsychotics (55.4%), respectively. Within-individual analysis showed that atypical antipsychotics (OR 0.70, 95% CI 0.55 to 0.89) and medication for addictive disorders (OR 0.66, 95% CI 0.47 to 0.93) were associated with reduced subsequent risk of coercive measures, compared with non-usage. Additionally, there was a reduced risk for recidivism associated with atypical antipsychotic use (OR 0.79, 95% CI 0.62 to 0.99) compared with periods of non-usage (figure 1). Periods of ADHD medication, compared with periods of non-usage, have point estimates suggesting an increased risk of both coercive measures (OR 1.22, 95% CI 0.84 to 1.76) and recidivism (OR 1.32, 95% CI 0.94 to 1.85), although not statistically significant.

### Antipsychotic treatment strategies

**Figure 2:**
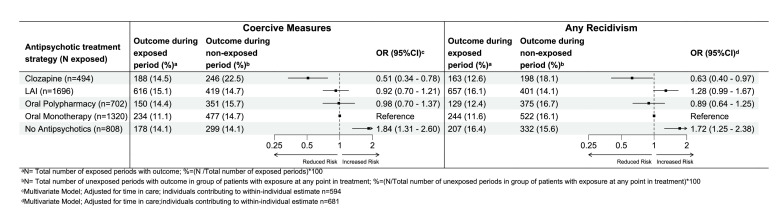
Associations between antipsychotic treatment strategies and coercive measures and recidivism. LAI, long-acting injectable.

When investigating different antipsychotic treatment strategies, LAI and oral monotherapy were the most frequently used treatment options, with 1696 and 1320 patients, respectively. Clozapine was associated with a reduced risk of coercive measures with an OR of 0.51 (95% CI 0.34 to 0.78) and recidivism with an OR of 0.63 (95% CI 0.40 to 0.97) in comparison to oral monotherapy. Both oral polypharmacy and LAI usage showed no significant risk difference relative to oral monotherapy for either outcome. No antipsychotic use was associated with an increased risk for both coercive measures and recidivism in comparison to antipsychotic monotherapy (figure 2).

### Specific antipsychotic agents

**Figure 3:**
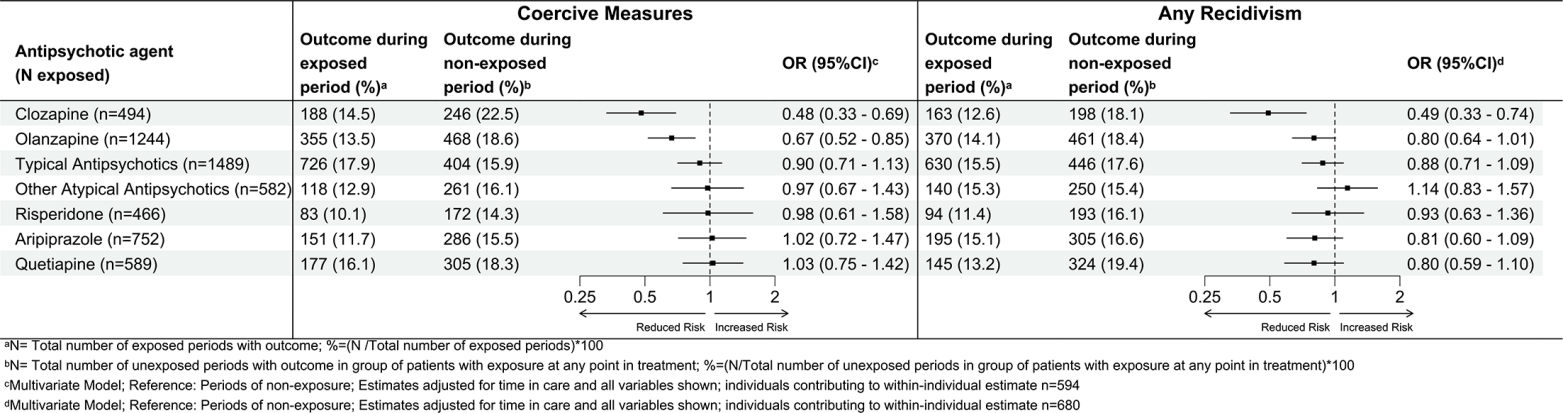
Associations between antipsychotic agents and coercive measures and recidivism.

Among specific antipsychotic agents, analyses showed that clozapine was associated with the largest reduction for subsequent coercive measures (OR 0.48, 95% CI 0.33 to 0.69), followed by olanzapine (OR 0.67, 95% CI 0.52 to 0.85), while the CIs were overlapping. For recidivism, periods of clozapine use (OR 0.49, 95% CI 0.33 to 0.74) were also associated with the largest risk reduction, followed by olanzapine (OR 0.80, 95% CI 0.64 to 1.01) and quetiapine (OR 0.80, 95% CI 0.59 to 1.10). However, the CIs overlapped in this comparison as well (figure 3).

### Secondary analyses

Subgroup analysis in patients with and without SUD showed differences in the direction of the associations for certain exposure-outcome combinations (online supplemental eTables 4–6). For example, antidepressant usage compared with non-usage in the group of patients with SUD was associated with an increased risk of subsequent coercive measures (OR 1.39, 95% CI 1.02 to 1.89), while the point estimate in the group without SUD suggests a decreased risk (OR 0.79, 95% CI 0.55 to 1.12), though with overlapping CIs (online supplemental eTable 4). Clozapine use was associated with a significant risk reduction for coercive measures and recidivism in comparison to oral monotherapy in the subgroup without SUD. In the subgroup with SUD, the point estimates suggest a similar association, however, with wider CIs (online supplemental eTable 5).

The association of pharmacotherapy and recidivism occurring during inpatient and outpatient care was compared (online supplemental eTables 7–9). During inpatient care periods, clozapine was associated with a reduced risk of recidivism both as a treatment strategy in comparison to oral monotherapy and as a specific agent. During outpatient periods, this association was, however, not observable (online supplemental eTables 8 and 9).

The sensitivity analysis excluding the first time period after admission resulted in similar risk estimates as the main analysis, however, with partially wider CIs (online supplemental eTables 10–12).

## Discussion

Using Swedish registry data and a self-controlled study design, we assessed the association of different pharmacological treatment regimens with the use of coercive measures and recidivism during FPC in 2690 patients. We found that atypical antipsychotics and medication for SUD were associated with a reduced risk of subsequent use of coercive measures. Further, atypical antipsychotics were associated with a reduced risk of subsequent recidivism. The use of oral polypharmacy and LAI showed no significant difference to oral monotherapy. Clozapine was found to be associated with the strongest risk reductions when considering different antipsychotic treatment strategies or specific antipsychotic agents.

To our knowledge, this is the first study to demonstrate that antipsychotics and SUD medications are associated with reductions in coercive measures, and that antipsychotics are associated with reduced recidivism, during FPC in a large national sample. Our results mirror those of real-world studies in other populations: periods of treatment with antipsychotics and medications for SUD have previously been associated with a reduced risk of violent crime in the population of persons who were released from prison.^6^ Similar associations have been observed for antipsychotics in the general psychiatric population of individuals receiving antipsychotics or mood stabilisers.^20^ Further, our findings are in line with previous studies reporting stronger risk reduction associated with clozapine and olanzapine compared with other antipsychotic agents.^8 21 22^ A previous study reported that among individuals with psychiatric disorders, the strongest risk reduction of criminal arrests and convictions was found during periods of clozapine use.^8^ This mirrors our results in which clozapine was associated with the strongest risk reduction of recidivism both among antipsychotic treatment strategies as well as specific antipsychotic agents. The side effect profile of clozapine, which includes an increased risk for agranulocytosis, requires regular blood monitoring and also closer clinical monitoring compared with other antipsychotics.^23^ This increased monitoring could partially explain why clozapine often shows better effectiveness than other antipsychotics, as non-adherence or symptom relapses are identified earlier and can be acted on. In the FPC setting, patients are, however, closely monitored independent of the medication they receive, so that additional monitoring due to clozapine is unlikely to fully explain the observed superiority of clozapine in its association with lower rates of coercive measures and recidivism in this context. In the presented sample, around 95% of patients who received clozapine at some point during FPC had a schizophrenia spectrum disorder (online supplemental eTable 13).

Other non-clozapine antipsychotics have previously been associated with a reduced risk of criminal arrests and convictions in individuals with psychotic disorders^8^; however, we only found a significant risk reduction of coercive measures with olanzapine. Olanzapine has been suggested to have more pronounced antiaggressive effects in comparison to other non-clozapine antipsychotics,^24^ as well as higher sedative properties, which might contribute to improved impulse control.^23^ The extent to which olanzapine is superior to other antipsychotics in reducing criminal and violent behaviour, and the mechanisms underlying this potential superiority, requires further investigation.

When comparing different treatment strategies of antipsychotics, oral polypharmacy and LAI use demonstrated no risk difference compared with oral monotherapy for coercive measures and any recidivism. These results go partially along with previous studies showing no clear evidence for the superiority or inferiority of antipsychotic polypharmacy over monotherapy regarding symptom improvement or relapse.^18 25^ LAI, however, has previously shown superiority to oral usage of most antipsychotics in non-FPC settings.^8 18^ These differences might reflect challenges with treatment adherence in the non-FPC settings, which can, to some extent, be mitigated through LAI usage.^26^ In contrast, the monitoring and control of patients within the FPC is on a much higher level, so that adherence is usually less of a problem.

ADHD medication stood out as a psychotropic medication class because it consistently produced point estimates suggesting an increased risk for the outcomes. These findings, however, did not reach statistical significance, and overinterpretation should be avoided. Nevertheless, there is an ongoing discussion regarding the safety of using ADHD medication in patients with psychotic disorders and in combination with antipsychotics,^27 28^ and treatment decisions should be made with caution in FPC. The comparison of patients with and without SUD showed that around one-third of the patients had an SUD diagnosis registered. The patients in the SUD group were characterised by a certain chronicity and severity of the disorder in this analysis, as only patients with the respective diagnoses were included, and not every patient with a history of substance abuse.^13^ The treatment of patients with a comorbid SUD represents a particular challenge due to interactions with other conditions and potentially elevated vulnerability for side effects. However, we found no statistical differences between the two groups, indicating a certain level of reassurance that clinical approaches work similarly in both groups for most medications. Differences in point estimate direction, as observed for instance in the use of antidepressants, as well as side effect profiles in the two groups, warrant nonetheless further investigation to allow for further conclusions.

The major strength of this study is the use of the national registry, allowing the analysis of nearly all individuals sentenced to FPC in Sweden since 2009. This makes the sample size of this study substantially larger than any previous studies on pharmacotherapy in FPC. Further, using a self-controlled study design reduced the bias of confounding, as all time-invariant confounders are adjusted for by the design.

There are, nevertheless, some limitations. First, the data format of the SNFPR was limited to cross-sectional information on medication use and retrospective period-level information about the outcome at every follow-up; the latter lending access only to information on the existence, but not on the frequency of an outcome between two follow-ups. Therefore, we were unable to examine the concurrent association between exposure and outcome and were restricted to only showing how medication use is associated with subsequent outcomes. As a consequence, exposure misclassification due to limited timing information cannot be ruled out. We assume, however, that these limitations in the data structure would dilute the strength of the associations, as this potential exposure misclassification would occur independently of subsequent outcome status.

Second, while the risk of confounding by indication has been reduced by the chosen study design, it cannot be completely ruled out, as a patient might receive stronger or more medications during periods with worse symptoms. The ‘true’ protective effect of the medication may therefore be underestimated in our analysis. Closely related is the risk of protopathic bias, in which early symptoms of the outcome cause treatment initiation. This bias is, however, likely to impact the results only to a minor extent, as the outcomes of interest are of an acute nature, and patients in FPC generally have a high baseline risk for the outcomes.

Third, the assumption of self-controlled designs that an outcome does not influence a subsequent exposure may not be fully met in this context.^12^ Psychotropic medication could be used or adjusted in response to violent or aggressive behaviour, resulting in coercive measures or recidivism. In the presented analysis, the recorded data points are, however, not dependent on the timing of exposure or outcome changes, reducing the immediate effect such a change would have on the concluded estimates. Moreover, excluding the first period after admission, a period that is at highest risk for violating this model assumption, did not result in considerable differences.

Fourth, information on other forms of treatment, such as behavioural and psychosocial treatments, was not available. Therefore, the effect of psychosocial treatments or their interaction with pharmacotherapy could not be assessed. Lastly, this study addresses only one dimension of the treatment outcomes of FPC. Other aspects, such as total length of FPC, general symptom reduction, ability to reintegrate into society and management of adverse effects of medications, are also of high clinical relevance.^7 29 30^ More high-quality research on treatment regimens in FPC is needed to adequately reflect these additional, sometimes competing, objectives of the treatment.

## Clinical implications

This study showed that atypical antipsychotics and SUD medications were associated with lower use of coercive measures, while atypical antipsychotics were additionally associated with reduced recidivism during FPC. Among antipsychotic agents, clozapine was associated with the strongest risk reduction for both coercive measures and recidivism, followed by olanzapine for coercive measures. These results may help inform treatment decisions in the management of risk behaviours. The findings are relevant in Sweden and in other countries that have a similar treatment system for criminal offenders with severe mental disorder, where the reduction of coercive measures and criminal behaviour is favourable.

## Supplementary material

10.1136/bmjment-2026-302515 online supplemental file 1

## Data Availability

Data may be obtained from a third party and are not publicly available.
