# Supplementary material for: Association of psychotropic medications with the use of coercive measures and recidivism during forensic psychiatric care: a Swedish nationwide register-based study
Source: BMJ Ment Health. 2026 Jun 17;29(1):e302515. doi: 10.1136/bmjment-2026-302515 (PMC13289076; doi:10.1136/bmjment-2026-302515)
Supplement: online supplemental file 1 [file bmjment-29-1-s001.docx]

**Supplementary Material**

[eTable 1: Categorization of typical and atypical antipsychotics 2](#_Toc230176184)

[eFigure 1: Illustration of the within-individual design for the exposure-outcome relationship. 3](#_Toc230176185)

[eMethods 1: Missingness 4](#_Toc230176186)

[eTable 2: Individuals with respective outcome by in- and outpatient care. 5](#_Toc230176187)

[eTable 3: Total number of periods with respective outcome by in- and outpatient care. 5](#_Toc230176188)

[eTable 4: Associations between major psychotropic medication and coercive measures and recidivism by SUD status 6](#_Toc230176189)

[eTable 5: Associations between antipsychotic treatment strategies and coercive measures and recidivism by SUD status 7](#_Toc230176190)

[eTable 6: Associations between antipsychotic agents and coercive measures and recidivism by SUD status 8](#_Toc230176191)

[eTable 7: Associations between major psychotropic medication and recidivism during in- and outpatient care. 9](#_Toc230176192)

[eTable 8: Associations between antipsychotic treatment strategies and recidivism during in- and outpatient care. 10](#_Toc230176193)

[eTable 9: Associations between antipsychotic agents and recidivism during in- and outpatient care. 11](#_Toc230176194)

[eTable 10: Associations between major psychotropic medication and coercive measures and recidivism after excluding the first observation period after admission. 12](#_Toc230176195)

[eTable 11: Associations between antipsychotic treatment strategies and coercive measures and recidivism after excluding the first observation period after admission. 13](#_Toc230176196)

[eTable 12: Associations between antipsychotic agents and coercive measures and recidivism after excluding the first observation period after admission. 14](#_Toc230176197)

[eTable 13: Characteristics of patients by clozapine status 15](#_Toc230176198)

# **eTable 1: Categorization of typical and atypical antipsychotics**

| **Typical Antipsychotics** | **Atypical Antipsychotics** |
| --- | --- |
| N05AA01 – Chlorpromazine  N05AF03 – Chlorprothixene  N05AF01 – Flupentixol  N05AB02 – Fluphenazine  N05AD01 – Haloperidol  N05AA02 – Levomepromazine  N05AH01 – Loxapine  N05AB03 – Perphenazine  N05AB04 – Prochlorperazine  N05AL01 – Sulpiride  N05AC02 – Thioridazine  N05AF05 – Zuclopenthixol  N05AA01 – Chlorpromazine | N05AL05 – Amisulpride  N05AX12 – Aripiprazole  N05AX15 – Cariprazine  N05AH02 – Clozapine  N05AE05 – Lurasidone  N05AD03 – Melperone  N05AH03 – Olanzapine  N05AX13 – Paliperidone  N05AH04 – Quetiapine  N05AX08 – Risperidone  N05AE03 – Sertindole  N05AL03 – Tiapride  N05AE04 – Ziprasidone |

# **eFigure 1: Illustration of the within-individual design for the exposure-outcome relationship.**


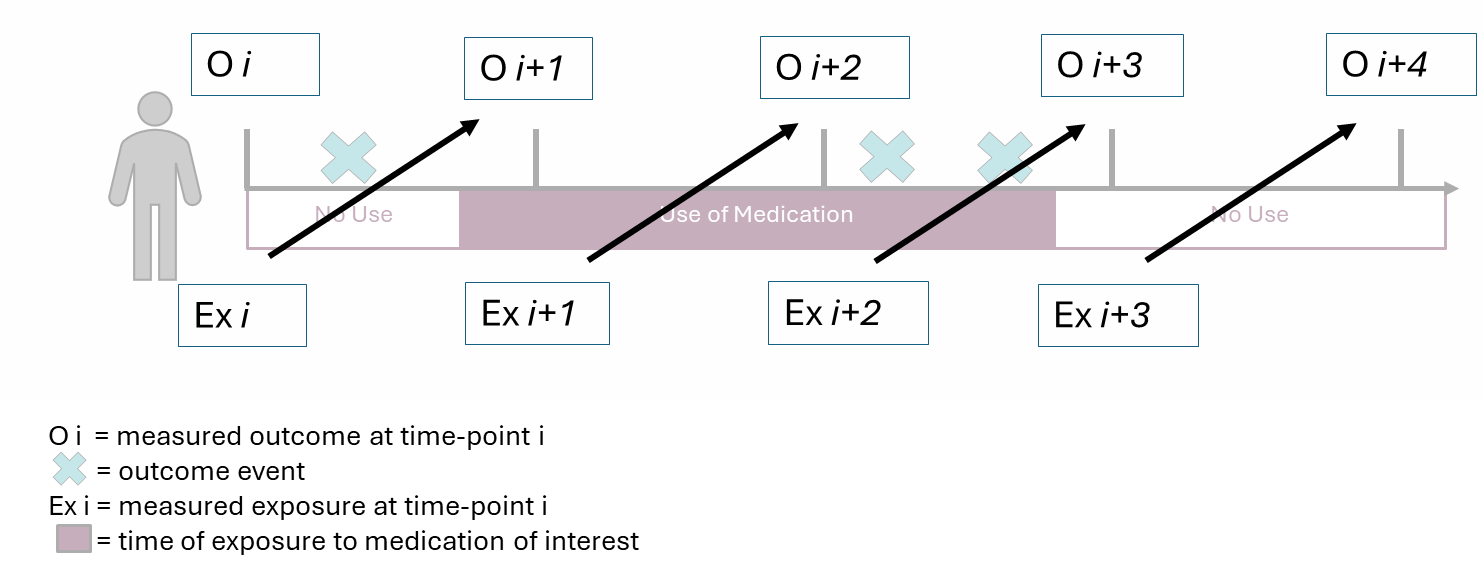


# **eMethods 1: Missingness**

For the analysis only periods with non-missing medication and outcome information were included. Medication was considered as missing, if an individual had a fully empty medication observation for a follow-up in SNFPR.

The outcomes were considered as missing, if the respective variables in SNFPR were empty for an observation. If an outcome is not possible in a time-period (e.g coercive measures while patient is in outpatient care), those respective time-periods were also excluded from the analysis, however they are not considered missing. The exact number of patients included in every analysis is noted in the description of the result tables.

The proportion of excluded time periods, due to missingness, is presented in the following table:

|  | **Proportion of missing observations %** (missing periods/total eligible periods) | **Proportion of individuals with >=1 missing observation** (individuals with >=1 missing observation /total eligible individuals) ^a^ |
| --- | --- | --- |
| **Medication** | 4.5% (602 of 13318) | 14.1% (379 of 2690) |
| **Coercive Measures ^b^** | 0.4% (40 of 10316) | 1.4% (37 of 2639) |
| **Recidivism** | 0.4% (54 of 13318) | 1.9% (50 of 2690) |
| *^a^ Individuals with missingness in one time-period are still included with non-missing time-periods in the analysis.*  *^b^ The total number of eligible periods and individuals for coercive measures is lower than for the other variables, because only periods not spent exclusively in outpatient care are considered eligible* | | |

# **eTable 2: Individuals with respective outcome by in- and outpatient care.**

|  | **Anytime**  **N (% of total)** | **Inpatient**  **N (% of total)** | **Outpatient**  **N (% of total)** |
| --- | --- | --- | --- |
| *Coercive Measure total* | 674 | 674 | - |
| Physical Restrain | 272 (40.4) | 272 (40.4) | - |
| Seclusion | 614 (91.1) | 614 (91.1) | - |
| Medication administration under physical restraint | 278 (41.2) | 278 (41.2) | - |
| *Recidivism Overall total* | 730 | 584 | 293 |
| Drug Related | 278 (38.1) | 197 (33.7) | 126 (43.0) |
| Violent | 317 (43.4) | 284 (48.6) | 50 (17.1) |
| Theft | 19 (2.6) | 13 (2.2) | 7 (2.4) |
| Damage/Vandalism | 60 (8.2) | 50 (8.6) | 11 (3.8) |
| Other type | 364 (49.9) | 271 (46.4) | 132 (45.1) |

# **eTable 3: Total number of periods with respective outcome by in- and outpatient care.**

|  | **Anytime**  **N (% of total)** | **Inpatient**  **N (% of total)** | **Outpatient**  **N (% of total)** |
| --- | --- | --- | --- |
| *Coercive Measure total* | 1410 | 1410 | - |
| Physical Restrain | 459 (32.6) | 459 (32.6) | - |
| Seclusion | 1273 (90.3) | 1273 (90.3) | - |
| Medication administration under physical restraint | 425 (30.1) | 425 (30.1) | - |
| *Recidivism total* | 1456 | 1092 | 445 |
| Drug Related | 463 (31.8) | 304 (27.8) | 186 (41.8) |
| Violent | 522 (37.9) | 476 (34.6) | 55 (12.4) |
| Theft | 22 (1.5) | 14 (1.3) | 8 (1.8) |
| Damage/Vandalism | 77 (5.3) | 66 (6.0) | 12 (2.7) |
| Other type | 536 (36.8) | 393 (36.0) | 173 (38.9) |

# **eTable 4: Associations between major psychotropic medication and coercive measures and recidivism by SUD status**

|  | **Subgroup: Patients with SUD Diagnosis  (N= 968)** | | **Subgroup: Patients without SUD Diagnosis (N= 1722)** | |
| --- | --- | --- | --- | --- |
| **Major classes of psychotropic medication** | **Coercive Measures OR (95%CI)^a^** | **Any Recidivism OR (95%CI)^b^** | **Coercive Measures OR (95%CI)^c^** | **Any Recidivism OR (95%CI)^d^** |
| Atypical Antipsychotics | 0.72 (0.53 - 0.99) | 0.78 (0.58 - 1.04) | 0.70 (0.48 - 1.01) | 0.81 (0.56 - 1.18) |
| Typical Antipsychotics | 0.78 (0.56 - 1.08) | 1.00 (0.75 - 1.33) | 1.07 (0.75 - 1.51) | 0.72 (0.51 - 1.03) |
| Antidepressants | 1.39 (1.02 - 1.89) | 1.24 (0.93 - 1.65) | 0.79 (0.55 - 1.12) | 0.69 (0.47 - 1.02) |
| Mood Stabilizers | 1.05 (0.69 - 1.58) | 1.12 (0.74 - 1.68) | 0.59 (0.34 - 1.02) | 0.59 (0.32 - 1.06) |
| ADHD Medication | 1.23 (0.80 - 1.89) | 1.24 (0.84 - 1.84) | 1.10 (0.54 - 2.24) | 1.46 (0.76 - 2.81) |
| Hypnotics and Sedatives | 0.82 (0.62 - 1.10) | 0.89 (0.69 - 1.15) | 1.18 (0.86 - 1.61) | 1.07 (0.79 - 1.45) |
| Antiepileptics | 0.93 (0.61 - 1.43) | 0.92 (0.65 - 1.29) | 1.44 (0.84 - 2.44) | 1.33 (0.76 - 2.32) |
| Opioids | 1.56 (0.45 - 5.39) | 1.07 (0.41 - 2.75) | 0.40 (0.12 - 1.35) | 1.67 (0.12 - 24.08) |
| Addiction Medication | 0.67 (0.44 - 1.01) | 0.78 (0.58 - 1.06) | 0.58 (0.31 - 1.10) | 1.20 (0.66 - 2.18) |
| *^a^ Individuals contributing to estimates n=285*  *^b^Individuals contributing to estimates n=354*  *^c^Individuals contributing to estimates n=309*  *^d^Individuals contributing to estimates n=325* | | | |  |

# **eTable 5: Associations between antipsychotic treatment strategies and coercive measures and recidivism by SUD status**

|  | **Subgroup: Patients with SUD Diagnosis (N= 968)** | | **Subgroup: Patients without SUD Diagnosis (N= 1722)** | |
| --- | --- | --- | --- | --- |
| **Antipsychotic Treatment Strategy** | **Coercive Measures OR (95%CI)^a^** | **Any Recidivism OR (95%CI)^b^** | **Coercive Measures OR (95%CI)^c^** | **Any Recidivism OR (95%CI)^d^** |
| Oral Monotherapy | 1 [Reference] | 1 [Reference] | 1 [Reference] | 1 [Reference] |
| Oral Polypharmacy | 0.85 (0.51 - 1.4) | 0.88 (0.56 - 1.37) | 1.11 (0.7 - 1.75) | 0.9 (0.53 - 1.51) |
| LAI | 1.03 (0.7 - 1.5) | 1.25 (0.9 - 1.72) | 0.82 (0.55 - 1.21) | 1.35 (0.86 - 2.12) |
| Clozapine | 0.61 (0.34 - 1.09) | 0.79 (0.46 - 1.36) | 0.41 (0.23 - 0.73) | 0.37 (0.18 - 0.77) |
| No Antipsychotics | 1.99 (1.28 - 3.11) | 1.55 (1.05 - 2.3) | 1.71 (0.99 - 2.93) | 2.06 (1.18 - 3.6) |
| *^a^ Individuals contributing to estimates n=285*  *^b^Individuals contributing to estimates n=354*  *^c^Individuals contributing to estimates n=309*  *^d^Individuals contributing to estimates n=327* | | | |  |

# **eTable 6: Associations between antipsychotic agents and coercive measures and recidivism by SUD status**

|  | **Subgroup: Patients with SUD Diagnosis**  **(N= 968)** | | **Subgroup: Patients without SUD Diagnosis**  **(N= 1722)** | |
| --- | --- | --- | --- | --- |
| **Antipsychotic agent** | **Coercive Measures OR (95%CI)^a^** | **Any Recidivism OR (95%CI)^b^** | **Coercive Measures OR (95%CI)^c^** | **Any Recidivism OR (95%CI)^d^** |
| Olanzapine | 0.63 (0.46 - 0.86) | 0.84 (0.63 - 1.12) | 0.71 (0.47 - 1.07) | 0.75 (0.51 - 1.10) |
| Aripiprazole | 0.86 (0.50 - 1.48) | 0.66 (0.45 - 0.96) | 1.28 (0.80 - 2.03) | 1.02 (0.64 - 1.61) |
| Clozapine | 0.54 (0.32 - 0.89) | 0.66 (0.41 - 1.06) | 0.40 (0.24 - 0.68) | 0.27 (0.13 - 0.54) |
| Quetiapine | 1.07 (0.74 - 1.55) | 0.79 (0.54 - 1.16) | 0.96 (0.53 - 1.75) | 0.72 (0.41 - 1.28) |
| Risperidone | 1.55 (0.79 - 3.02) | 1.02 (0.61 - 1.70) | 0.68 (0.35 - 1.32) | 0.82 (0.46 - 1.44) |
| Typical Antipsychotics | 0.77 (0.57 - 1.05) | 1.02 (0.77 - 1.35) | 1.05 (0.73 - 1.50) | 0.71 (0.50 - 1.01) |
| Other Atypical Antipsychotics | 0.83 (0.48 - 1.45) | 1.06 (0.72 - 1.57) | 1.16 (0.69 - 1.93) | 1.30 (0.77 - 2.17) |
| *^a^ Individuals contributing to estimates n=285*  *^b^Individuals contributing to estimates n=354*  *^c^Individuals contributing to estimates n=309*  *^d^Individuals contributing to estimates n=326* | | | |  |

# **eTable 7: Associations between major psychotropic medication and recidivism during in- and outpatient care.**

|  | **Recidivism in Inpatient Care** | | | **Recidivism in Outpatient Care** | | | |
| --- | --- | --- | --- | --- | --- | --- | --- |
| **Major classes of psychotropic medication (n=exposed)** | **Outcome during exposed period (%)^a^** | **Outcome during unexposed period (%)^b^** | **OR (95%CI)^c^** | **Outcome during exposed period (%)^a^** | **Outcome during unexposed period (%)^b^** | **OR (95%CI)^d^** | |
| Atypical Antipsychotics (n=2257) | 649 (10) | 286 (13.4) | 0.78 (0.61 – 1.00) | 289 (4.4) | 55 (2.6) | 1.10 (0.64 - 1.89) | |
| Typical Antipsychotics (n=1489) | 487 (12) | 329 (13) | 0.91 (0.72 - 1.15) | 178 (4.4) | 91 (3.6) | 0.98 (0.59 - 1.62) | |
| Antidepressants (n=1326) | 380 (10.8) | 245 (11.1) | 0.96 (0.73 - 1.26) | 155 (4.4) | 49 (2.2) | 1.03 (0.60 - 1.75) | |
| Mood Stabilizers (n=675) | 201 (11.4) | 190 (14.7) | 0.86 (0.59 - 1.24) | 79 (4.5) | 30 (2.3) | 1.22 (0.57 - 2.63) | |
| ADHD Medication (n=341) | 155 (18.9) | 154 (16.6) | 1.25 (0.85 - 1.83) | 47 (5.7) | 22 (2.4) | 1.37 (0.70 - 2.68) | |
| Hypnotics and Sedatives (n=1605) | 474 (12.9) | 331 (11.1) | 0.92 (0.74 - 1.14) | 178 (4.8) | 116 (3.9) | 0.87 (0.61 - 1.25) | |
| Antiepileptics (n=447) | 157 (14.9) | 181 (14.6) | 1.26 (0.91 - 1.76) | 47 (4.5) | 27 (2.2) | 1.20 (0.59 - 2.41) | |
| Opioids (n=81) | 14 (14.7) | 32 (10.5) | 1.40 (0.40 - 4.97) | 9 (9.5) | 5 (1.6) | 0.66 (0.13 - 3.25) | |
| Addiction Medication (n=523) | 82 (8.7) | 200 (12.3) | 0.77 (0.54 - 1.09) | 76 (8.1) | 74 (4.5) | 0.77 (0.49 - 1.22) | |
| *^a^N= Total number of exposed periods with outcome; %=(N /Total number of exposed periods)*100*  *^b^N= Total number of unexposed periods with outcome in group of patients with exposure at any point in treatment; %=(N/Total number of unexposed periods in group of patients with exposure at any point in treatment)*100*  *^c^Multivariate Model; adjusted for time in care and for all shown variables; Individuals contribution to within-individual estimate n=529*  *^d^Multivariate Model; adjusted for time in care and for all shown variables; Individuals contribution to within-individual estimate n=253* | | | | | | |  |

# **eTable 8: Associations between antipsychotic treatment strategies and recidivism during in- and outpatient care.**

|  | **Any Recidivism in Inpatient Care** | | | **Any Recidivism in Outpatient Care** | | | |
| --- | --- | --- | --- | --- | --- | --- | --- |
| **Antipsychotic Treatment Strategy (n= exposed)** | **Outcome during exposed period (%)^a^** | **Outcome during unexposed period (%)^b^** | **OR (95%CI)^c^** | **Outcome during exposed period (%)^a^** | **Outcome during unexposed period (%)^b^** | **OR (95%CI)^d^** | |
| Oral Monotherapy (n=1320) | 192 (9.1) | 363 (11.2) | 1 [Reference] | 62 (2.9) | 88 (2.7) | 1 [Reference] | |
| Oral Polypharmacy (n=702) | 102 (9.8) | 259 (11.6) | 0.92 (0.63 - 1.36) | 32 (3.1) | 71 (3.2) | 0.82 (0.39 - 1.76) | |
| LAI (n=1696) | 474 (11.6) | 299 (10.5) | 1.20 (0.90 - 1.60) | 225 (5.5) | 82 (2.9) | 1.23 (0.64 - 2.34) | |
| Clozapine (n=494) | 117 (9) | 169 (15.4) | 0.52 (0.32 - 0.86) | 54 (4.2) | 20 (1.8) | 1.36 (0.49 - 3.77) | |
| No Antipsychotics Use (n=808) | 164 (13) | 249 (11.7) | 1.53 (1.08 - 2.18) | 55 (4.4) | 56 (2.6) | 1.54 (0.66 - 3.58) | |
| *^a^N= Total number of exposed periods with outcome; %=(N /Total number of exposed periods)*100*  *^b^N= Total number of unexposed periods with outcome in group of patients with exposure at any point in treatment; %=(N/Total number of unexposed periods in group of patients with exposure at any point in treatment)*100*  *^c^Multivariate Model; adjusted for time in care; Individuals contribution to within-individual estimate n=530*  *^d^Multivariate Model; adjusted for time in care; Individuals contribution to within-individual estimate n=254* | | | | | | |  |

# **eTable 9: Associations between antipsychotic agents and recidivism during in- and outpatient care.**

|  | **Any Recidivism in Inpatient Care** | | | **Any Recidivism in Outpatient Care** | | | |
| --- | --- | --- | --- | --- | --- | --- | --- |
| **Antipsychotic agent (n =exposed)** | **Outcome during exposed period (%)^a^** | **Outcome during unexposed period (%)^b^** | **OR (95%CI)^c^** | **Outcome during exposed period (%)^a^** | **Outcome during unexposed period (%)^b^** | **OR (95%CI)^d^** | |
| Olanzapine (n=1244) | 283 (10.8) | 343 (13.7) | 0.79 (0.61 - 1.03) | 106 (4.0) | 77 (3.1) | 0.70 (0.43 - 1.16) | |
| Aripiprazole (n=752) | 132 (10.2) | 234 (12.7) | 0.82 (0.58 - 1.17) | 68 (5.3) | 41 (2.2) | 0.86 (0.44 - 1.71) | |
| Clozapine (n=494) | 117 (9.0) | 169 (15.4) | 0.44 (0.28 - 0.69) | 54 (4.2) | 20 (1.8) | 1.07 (0.44 - 2.60) | |
| Quetiapine (n=589) | 112 (10.2) | 229 (13.7) | 0.83 (0.58 - 1.18) | 41 (3.7) | 53 (3.2) | 0.77 (0.39 - 1.51) | |
| Risperidone (n=466) | 64 (7.8) | 139 (11.6) | 0.97 (0.63 - 1.49) | 39 (4.7) | 41 (3.4) | 1.14 (0.53 - 2.45) | |
| Typical Antipsychotics (n=1489) | 487 (12) | 329 (13) | 0.91 (0.72 - 1.16) | 178 (4.4) | 91 (3.6) | 0.95 (0.58 - 1.55) | |
| Other Atypical Antipsychotics (n=582) | 102 (11.1) | 191 (11.8) | 1.34 (0.94 - 1.92) | 46 (5.0) | 41 (2.5) | 0.62 (0.28 - 1.37) | |
| *^a^N= Total number of exposed periods with outcome; %=(N /Total number of exposed periods)*100*  *^b^N= Total number of unexposed periods with outcome in group of patients with exposure at any point in treatment; %=(N/Total number of unexposed periods in group of patients with exposure at any point in treatment)*100*  *^c^Multivariate Model; adjusted for time in care and for all shown variables; Individuals contribution to within-individual estimate n=529*  *^d^Multivariate Model; adjusted for time in care and for all shown variables; Individuals contribution to within-individual estimate n=254* | | | | | | |  |

# **eTable 10: Associations between major psychotropic medication and coercive measures and recidivism after excluding the first observation period after admission.**

|  | **Coercive Measures** | | | **Any Recidivism** | | | |
| --- | --- | --- | --- | --- | --- | --- | --- |
| **Major classes of psychotropic medication (n=exposed)** | **Outcome during exposed period (%)^a^** | **Outcome during unexposed period (%)^b^** | **OR (95%CI)^c^** | **Outcome during exposed period (%)^a^** | **Outcome during unexposed period (%)^b^** | **OR (95%CI)^d^** | |
| Atypical Antipsychotics (n=1886) | 623 (12.9) | 265 (18.7) | 0.66 (0.50 - 0.88) | 704 (14.6) | 259 (18.3) | 0.80 (0.61 - 1.03) | |
| Typical Antipsychotics (n=1172) | 519 (17.3) | 288 (17.4) | 0.80 (0.60 - 1.06) | 494 (16.5) | 317 (19.2) | 0.87 (0.67 - 1.12) | |
| Antidepressants (n=1108) | 386 (14.1) | 231 (16.1) | 1.25 (0.97 - 1.63) | 398 (14.5) | 236 (16.4) | 0.92 (0.71 - 1.21) | |
| Mood Stabilizers (n=550) | 254 (18.8) | 186 (22.4) | 0.83 (0.56 - 1.23) | 207 (15.3) | 157 (18.9) | 0.84 (0.56 - 1.28) | |
| ADHD Medication (n=289) | 126 (18.6) | 122 (19.5) | 1.27 (0.82 - 1.94) | 146 (21.5) | 134 (21.4) | 1.15 (0.79 - 1.66) | |
| Hypnotics and Sedatives (n=1283) | 414 (15.3) | 304 (14.6) | 0.98 (0.76 - 1.27) | 476 (17.6) | 356 (17.1) | 0.89 (0.71 - 1.12) | |
| Antiepileptics (n=380) | 160 (19.2) | 206 (23.7) | 1.14 (0.75 - 1.72) | 147 (17.6) | 173 (19.9) | 1.03 (0.72 - 1.47) | |
| Opioids (n=66) | 12 (17.1) | 40 (17.3) | 0.92 (0.33 - 2.53) | 18 (25.7) | 34 (14.7) | 1.71 (0.61 - 4.79) | |
| Addiction Medication (n=450) | 84 (10.7) | 206 (18) | 0.50 (0.34 - 0.75) | 122 (15.5) | 227 (19.8) | 0.77 (0.57 - 1.04) | |
| *^a^N= Total number of exposed periods with outcome; %=(N /Total number of exposed periods)*100*  *^b^N= Total number of unexposed periods with outcome in group of patients with exposure at any point in treatment; %=(N/Total number of unexposed periods in group of patients with exposure at any point in treatment)*100*  *^c^Multivariate Model; adjusted for time in care and for all shown variables; Individuals contribution to within-individual estimate n= 413*  *^d^Multivariate Model; adjusted for time in care and for all shown variables; Individuals contribution to within-individual estimate n= 536* | | | | | | |  |

# **eTable 11: Associations between antipsychotic treatment strategies and coercive measures and recidivism after excluding the first observation period after admission.**

|  | **Coercive Measures** | | | **Any Recidivism** | | | |
| --- | --- | --- | --- | --- | --- | --- | --- |
| **Antipsychotic Treatment Strategy (n= exposed)** | **Outcome during exposed period (%)^a^** | **Outcome during unexposed period (%)^b^** | **OR (95%CI)^c^** | **Outcome during exposed period (%)^a^** | **Outcome during unexposed period (%)^b^** | **OR (95%CI)^d^** | |
| Oral Monotherapy (n=969) | 148 (10.6) | 307 (14.2) | 1 [Reference] | 172 (12.3) | 341 (15.8) | 1 [Reference] | |
| Oral Polypharmacy (n=478) | 93 (14.0) | 226 (16.7) | 0.91 (0.59 - 1.40) | 89 (13.4) | 249 (18.4) | 0.84 (0.55 - 1.27) | |
| LAI (n=1426) | 466 (14.9) | 290 (15.2) | 0.91 (0.65 - 1.29) | 544 (17.4) | 293 (15.4) | 1.24 (0.91 - 1.71) | |
| Clozapine (n=442) | 155 (14.0) | 154 (21.1) | 0.69 (0.41 - 1.16) | 138 (12.4) | 139 (19.0) | 0.68 (0.41 - 1.13) | |
| No Antipsychotics (n=609) | 123 (13.1) | 206 (15.0) | 1.80 (1.18 - 2.73) | 154 (16.4) | 245 (17.8) | 1.59 (1.08 - 2.33) | |
| *^a^N= Total number of exposed periods with outcome; %=(N /Total number of exposed periods)*100*  *^b^N= Total number of unexposed periods with outcome in group of patients with exposure at any point in treatment; %=(N/Total number of unexposed periods in group of patients with exposure at any point in treatment)*100*  *^c^Multivariate Model; adjusted for time in care; Individuals contribution to within-individual estimate n=414*  *^d^Multivariate Model; adjusted for time in care; Individuals contribution to within-individual estimate n=539* | | | | | | |  |

# **eTable 12: Associations between antipsychotic agents and coercive measures and recidivism after excluding the first observation period after admission.**

|  | **Coercive Measures** | | | **Any Recidivism** | | | |
| --- | --- | --- | --- | --- | --- | --- | --- |
| **Antipsychotic agent (n =exposed)** | **Outcome during exposed period (%)^a^** | **Outcome during unexposed period (%)^b^** | **OR (95%CI)^c^** | **Outcome during exposed period (%)^a^** | **Outcome during unexposed period (%)^b^** | **OR (95%CI)^d^** | |
| Olanzapine (n=932) | 238 (13.1) | 321 (19.4) | 0.62 (0.45 - 0.84) | 280 (15.5) | 315 (19.0) | 0.77 (0.59 - 1.00) | |
| Aripiprazole (n=613) | 121 (12.0) | 207 (16.6) | 1.06 (0.70 - 1.59) | 159 (15.8) | 217 (17.4) | 0.79 (0.55 - 1.14) | |
| Clozapine (n=442) | 155 (14.0) | 154 (21.1) | 0.65 (0.41 - 1.02) | 138 (12.4) | 139 (19.0) | 0.53 (0.33 - 0.83) | |
| Quetiapine (n=444) | 126 (15.9) | 192 (17.7) | 0.95 (0.64 - 1.40) | 100 (12.6) | 218 (20.1) | 0.66 (0.46 - 0.95) | |
| Risperidone (n=342) | 50 (8.5) | 128 (17.8) | 0.85 (0.50 - 1.44) | 68 (11.6) | 132 (18.3) | 0.78 (0.49 - 1.24) | |
| Typical Antipsychotics (n=1172) | 519 (17.3) | 288 (17.4) | 0.84 (0.63 - 1.11) | 494 (16.5) | 317 (19.2) | 0.85 (0.66 - 1.09) | |
| Other Atypical Antipsychotics (n=486) | 93 (13.0) | 187 (16.5) | 1.09 (0.69 - 1.74) | 118 (16.4) | 187 (16.5) | 1.06 (0.73 - 1.54) | |
| *^a^N= Total number of exposed periods with outcome; %=(N /Total number of exposed periods)*100*  *^b^N= Total number of unexposed periods with outcome in group of patients with exposure at any point in treatment; %=(N/Total number of unexposed periods in group of patients with exposure at any point in treatment)*100*  *^c^Multivariate Model; adjusted for time in care and for all shown variables; Individuals contribution to within-individual estimate n=414*  *^d^Multivariate Model; adjusted for time in care and for all shown variables; Individuals contribution to within-individual estimate n=538* | | | | | | |  |

# **eTable 13: Characteristics of patients by clozapine status.**

|  | **No clozapine** | **Clozapine***^a^* | **p***^b^* |
| --- | --- | --- | --- |
| n | 2196 | 494 |  |
| Sex Male n (%) | 1814 (82.6) | 411 (83.2) | 0.855 |
| Age at first admission in years median (IQR) | 34.00 (27.00, 46.00) | 31.00 (25.00, 39.75) | <0.001 |
| Place of Birth Sweden n (%) | 1398 (63.7) | 338 (68.4) | 0.052 |
| Diagnoses |  |  |  |
| Schizophrenia Spectrum Disorder n (%) | 1612 (73.4) | 471 (95.3) | <0.001 |
| Substance Use Disorder n (%) | 755 (34.4) | 213 (43.1) | <0.001 |
| Bipolar Disorder n (%) | 211 (9.6) | 18 (3.6) | <0.001 |
| Recurrent Depressive Disorder n (%) | 67 (3.1) | 4 (0.8) | 0.008 |
| ADHD n (%) | 343 (15.6) | 71 (14.4) | 0.532 |
| Disorders of adult personality and behaviour n (%) | 595 (27.1) | 105 (21.3) | 0.009 |
| Autism n (%) | 432 (19.7) | 90 (18.2) | 0.500 |
| Intellectual Disability n (%) | 360 (16.4) | 76 (15.4) | 0.630 |
| *^a^ Clozapine use at any time during forensic psychiatric care*  *^b^ Chi-square test for categorical variables, and Mann-Whitney U test for continuous variables* | | | |
